# Developing a Novel Genomic Biomarker for the Early Detection of Neurodegeneration

**DOI:** 10.1002/alz70856_099024

**Published:** 2025-12-24

**Authors:** Martyn Frith, Barry Chioza, Rosemary Bamford, Eilis Hannon, Timothy Harrower, Jonathan Mill, Emma Dempster

**Affiliations:** ^1^ University of Exeter, Exeter, Devon, United Kingdom; ^2^ University of Exeter, Exeter, United Kingdom; ^3^ Univeristy of Exeter, Exeter, United Kingdom

## Abstract

**Background:**

Despite the large and growing global burden of neurodegenerative diseases, it is not currently possible to detect neurodegeneration before irreversible damage to the brain occurs, as this is the point at which clinical symptoms manifest. There is, therefore, an unmet need for a reliable and accurate clinical test that is capable of detecting neurodegeneration before the brain suffers permanent damage. Our aim is to leverage epigenetic profiling technology to develop a sequencing‐based assay that can detect cell‐free DNA (cfDNA) originating from degenerating neuronal tissue in human plasma samples as a biomarker for neurodegeneration.

**Method:**

Blood samples from a large cohort of neurodegenerative disease patients and from age‐ and sex‐matched controls are currently being collected. cfDNA will be directly sequenced using the Nanopore sequencing platform for native detection of cfDNA epigenetic modifications. A novel cell type deconvolution algorithm designed to handle to shortcomings of cfDNA sequencing data, Ranked Beta Binomial (RBB), will be used to detect neuron‐derived cfDNA fragments in plasma samples. Based on the results of cell type deconvolution we will assess the capability of this assay for detecting neurodegeneration using sensitivity and specificity analyses.

**Result:**

RBB deconvolutes cell type proportions from both *in silico* cfDNA mixtures and spike‐in control cfDNA samples with known proportions of neuron‐derived cfDNA with high accuracy, even when the read depth of data and the proportions neuron‐derived cfDNA in the sample are both low. Furthermore, RBB is capable of detecting neuron‐derived cfDNA fractions with high accuracy when deconvoluting cell type proportions from cfDNA sequencing data derived from real human plasma samples.

**Conclusion:**

While preliminary results for detection of neuron‐derived cfDNA as a biomarker neurodegeneration are promising, further development of cell type deconvolution methods and testing of diagnostic capability in patient populations are required in order to draw conclusions about the clinical utility of this assay.

